# Effects of Tillage Practices and Plastic Film Mulching on Soil Temperature, Dry Matter Production, and Yield Formation of Maize in a Cool Region

**DOI:** 10.3390/plants15203175

**Published:** 2026-10-16

**Authors:** Danhui Zhang, Fei Li, Chen Xu, Yangming Guo, Lihua Zhang, Ning Sun, Chang Peng, Hongxiang Zhao, Shaofeng Bian

**Affiliations:** 1 Faculty of Agronomy, Jilin Agricultural University, Changchun 130118, China; 20240739@mails.jlau.edu.cn (D.Z.); guoyangming1996@mails.jlau.edu.cn (Y.G.); 2 Institute of Agricultural Resources and Environment, Jilin Academy of Agriculture Sciences, Changchun 130033, China; lifei@jaas.com.cn (F.L.); xuchen@jaas.com.an (C.X.); zhanglihua@jaas.com.cn (L.Z.); sunning1984@jaas.com.cn (N.S.); pengchang@jaas.com.cn (C.P.); 3 Multidisciplinary, Observation and Research Station, Ministry of Agriculture and Rural Affairs, Taonan 137100, China

**Keywords:** cool-climate regions, maize, cropping systems, plastic mulching, soil temperature, dry matter accumulation, yield

## Abstract

Limited thermal resources in the cool regions of Northeast China restrict maize growth and yield formation. A two-year field experiment was conducted in Dunhua, Jilin Province, China, during 2024–2025 using four tillage practices—deep rotary tillage (SX), deep harrowing (SB), deep plowing (SF), and subsurface rotary tillage (QX)—combined with plastic film mulching or no mulching. Soil temperature, leaf area index (LAI), photosynthetic characteristics, dry matter accumulation, and grain yield were evaluated. Mulching increased soil temperature by 1.27–6.37 °C in 2024 and 1.37–5.20 °C in 2025, promoted canopy development, and improved photosynthetic performance. It also increased dry matter accumulation at V8 by 61.42–87.31% and enhanced post-silking dry matter accumulation. Grain yield increased by 7.93–10.51% in 2024 and 4.94–10.27% in 2025. Deep rotary tillage with mulching (SXC_1_) produced the highest yield in both years. Correlation analysis showed that soil temperature, LAI, and post-silking dry matter accumulation were closely associated with yield. Overall, deep rotary tillage combined with plastic film mulching improved the soil thermal environment, dry matter production, and maize yield in cool regions.

## 1. Introduction

Maize (Zea mays L.) is one of the most important cereal crops worldwide, and its growth, development, and yield formation are highly dependent on suitable thermal conditions. As a typical C_4_ crop, maize is relatively sensitive to low temperatures, particularly from sowing to the early vegetative growth stage. Low-temperature stress can inhibit seedling establishment, root development, and leaf expansion, and can further reduce photosynthetic capacity by impairing photosystem activity, carbon assimilation, and antioxidant metabolism [1,2,3]. These constraints are particularly pronounced in the cool maize-growing regions of Northeast China, where relatively low spring temperatures, limited thermal resources, and a short frost-free period often restrict early maize growth and subsequent dry matter production. Therefore, in cool regions characterized by slow spring warming, insufficient effective accumulated temperature, and a relatively short growing season, improving soil thermal conditions and promoting early maize growth through appropriate agronomic practices is essential for achieving stable and high yields.

Tillage practice is an important means of regulating soil structure and the crop rhizosphere environment. Different tillage practices can alter soil bulk density, pore structure, water storage, and root spatial distribution, thereby affecting canopy photosynthesis, dry matter accumulation, and grain formation [4]. A meta-analysis of field experiments conducted across different regions of China showed that deep tillage generally increased maize yield, although its effects were modulated by climate, soil type, and management conditions [5]. Under plastic film mulching systems, tillage practices such as rotary tillage and plowing can also markedly alter root distribution, nitrogen uptake, and translocation, resulting in different yield responses [6]. In recent years, combining reduced plastic film mulching with minimum tillage or no-tillage has been shown to improve soil water use and soil quality [7], while appropriate conservation tillage can promote maize production by maintaining root activity, leaf photosynthetic capacity, and canopy function [8,9,10]. Plastic film mulching is also an important agronomic practice for improving soil water and thermal conditions in cool and water-limited regions. Mulching can reduce soil evaporation, modify soil heat exchange, and increase soil temperature during the early growth stages, thereby improving maize root growth and water and nitrogen use [11,12]. Multi-year field experiments have shown that both biodegradable plastic film and conventional polyethylene film provide effective soil warming and moisture conservation during the early growth period and can increase leaf area index, aboveground biomass, and yield [13]. Meanwhile, biodegradable plastic film has potential advantages in reducing the risk of residual plastic accumulation in agricultural soils [14,15,16]. However, the agronomic performance of biodegradable film is closely related to its degradation process. Premature degradation may weaken soil heat and moisture conservation, thereby affecting water availability during grain filling and ultimately reducing yield [17]. Biodegradable films made from different materials also differ markedly in their effects on soil functions and maize productivity [18,19,20], indicating that their yield benefits are strongly dependent on environmental conditions and film properties. The soil water and thermal effects induced by plastic film mulching can further influence plant physiological processes. Recent studies have shown that plastic film mulching can promote root development and maintain a larger green leaf area and higher net photosynthetic rate, thereby increasing aboveground biomass and yield [21]. Optimizing the ridge–furrow mulching ratio can promote LAI development and dry matter accumulation by improving soil water and thermal conditions before silking [22]. The combination of plastic film mulching with appropriate nutrient management can also simultaneously enhance net photosynthetic rate, stomatal conductance, transpiration rate, and photochemical efficiency, ultimately promoting yield formation [23]. The yield-enhancing effect of plastic film mulching is attributable not only to improved soil water availability but also to enhanced crop transpiration, aboveground biomass accumulation, and harvest index [24]. Meta-analyses and recent long-term field experiments have further confirmed that the yield response to plastic film mulching is jointly regulated by temperature, precipitation, soil properties, and tillage management [25,26,27].

Maize grain yield ultimately depends on the coordination among the canopy photosynthetic “source”, dry matter accumulation and translocation, and grain “sink” capacity. High-yielding maize generally exhibits an appropriate leaf area index (LAI) at silking, strong post-silking dry matter production, and more efficient assimilate partitioning [28]. As maize yield increases, post-silking dry matter accumulation becomes increasingly important for final yield formation [29]. Enhanced post-silking accumulation of dry matter and nitrogen can significantly improve kernel number and grain weight, thereby promoting grain yield formation [30]. However, previous studies have mainly focused on single mulching practices, tillage measures, or water and fertilizer management in semi-arid or arid regions, whereas the continuous response of canopy development, photosynthetic characteristics, post-silking dry matter production, and yield formation to combinations of different tillage practices and plastic mulching through changes in soil temperature remains insufficiently understood in the cool regions of Northeast China. Based on this knowledge gap, we hypothesized that plastic mulching could alleviate thermal limitations during the early growth stages of maize in cool regions and interact synergistically with appropriate tillage practices, thereby promoting canopy establishment and leaf photosynthetic performance, enhancing post-silking dry matter production, and ultimately increasing grain yield. Therefore, a two-year field experiment was conducted using combinations of different tillage practices and plastic mulching to systematically evaluate their effects on soil temperature, leaf area index, leaf photosynthetic characteristics, dry matter accumulation, post-silking dry matter accumulation, grain yield, and yield-related traits of maize in a cool region, as well as the relationships between key physiological traits and grain yield. Compared with previous studies that mainly focused on single tillage measures or mulching effects, the present study further provides an integrated evaluation of four tillage practices combined with plastic mulching from the perspective of the continuous responses of soil temperature, canopy development, photosynthetic characteristics, dry matter production, and yield formation. This study aimed to clarify the coordinated effects of different tillage practices and plastic mulching on the soil thermal environment, biomass production, and yield formation of maize, thereby providing a theoretical basis for optimizing high-yield, high-efficiency, and sustainable maize cultivation systems in cool regions.

## 2. Results

### 2.1. Effects of Tillage Practices and Plastic Film Mulching on Soil Temperature

**Figure 1:**
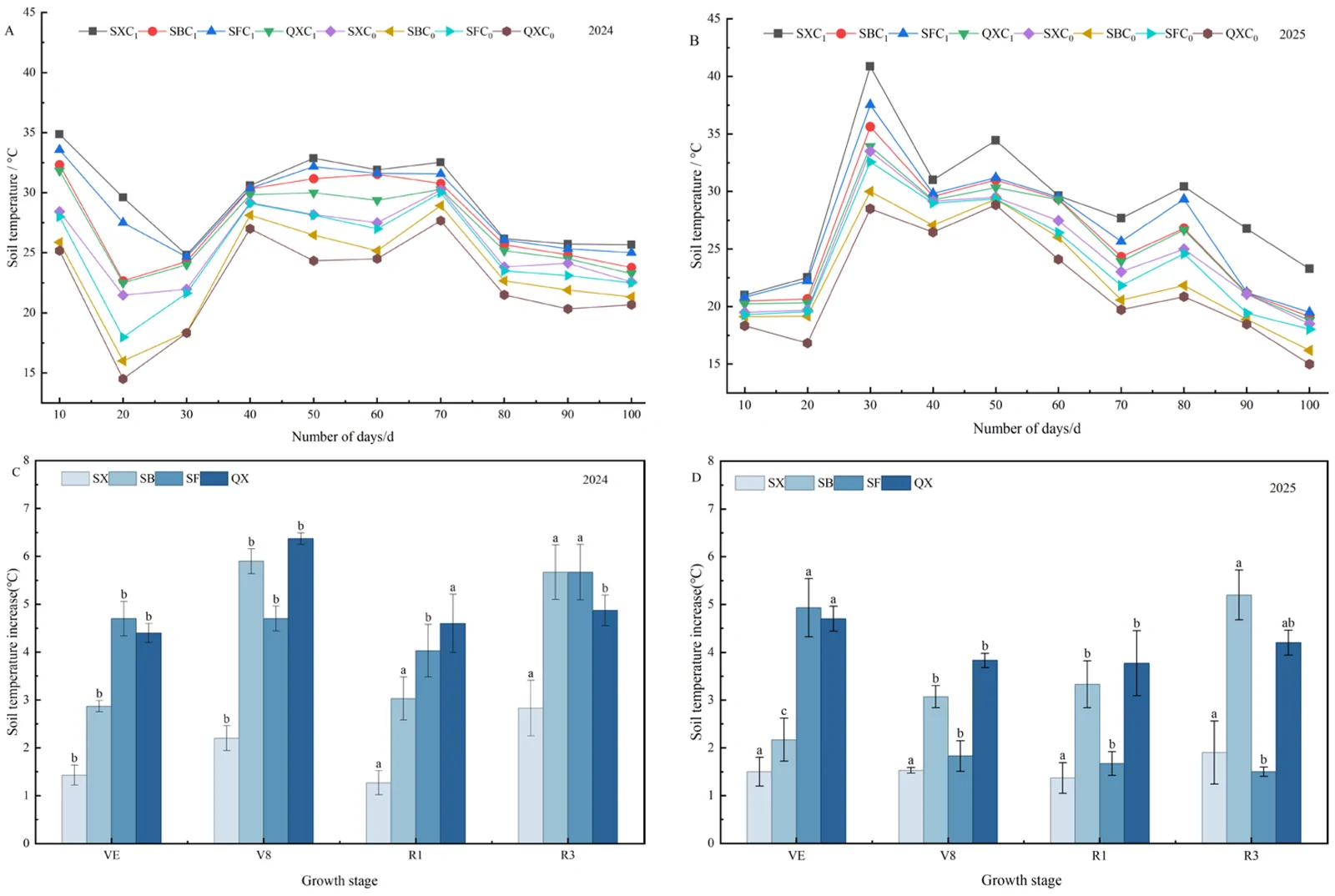
Soil temperature and warming magnitude under different tillage practices and plastic film mulching treatments. (**A**) Changes in soil temperature under different tillage practices and plastic film mulching treatments in 2024; (**B**) changes in soil temperature under different tillage practices and plastic film mulching treatments in 2025; (**C**) soil warming magnitude under different tillage practices at different growth stages in 2024; (**D**) soil warming magnitude under different tillage practices at different growth stages in 2025. Data are presented as the mean ± standard deviation (SD). The warming magnitude was calculated as $\Delta T=T_{C_{1}}-T_{C_{0}}$. Different lowercase letters indicate significant differences among tillage practices within the same growth stage at *p* < 0.05.

As shown in Figure 1, soil temperature under all treatments exhibited stage-dependent fluctuations in both 2024 and 2025, with the mulched treatments (C_1_) generally showing higher soil temperatures than the corresponding non-mulched treatments (C_0_). In 2024, the warming magnitude ranged from 1.27 to 6.37 °C. The greatest increase was observed at the milk stage (R3), where SBC_1_ increased soil temperature by 6.37 °C compared with SBC_0_. At the V8 stage, SBC_1_ and QXC_1_ increased soil temperature by 5.90 and 5.67 °C, respectively, relative to their corresponding non-mulched treatments. In 2025, the warming magnitude ranged from 1.37 to 5.20 °C. At the V8 stage, SXC_1_ increased soil temperature by 4.93 °C compared with SXC_0_, and this warming magnitude was significantly greater than that observed under the other tillage practices (*p* < 0.05). At the silking stage (R1), QXC_1_ increased soil temperature by 5.20 °C compared with QXC_0_, which was also significantly greater than the increases under the other treatments (*p* < 0.05). In contrast, no significant differences in warming magnitude among tillage practices were observed at the emergence stage (VE) or R3 stage (*p* > 0.05). Overall, these results indicate that the soil-warming effect of plastic film mulching was jointly influenced by growth stage, tillage practice, and year.

### 2.2. Effects of Tillage Practices and Plastic Film Mulching on Maize Leaf Area Index

**Figure 2:**
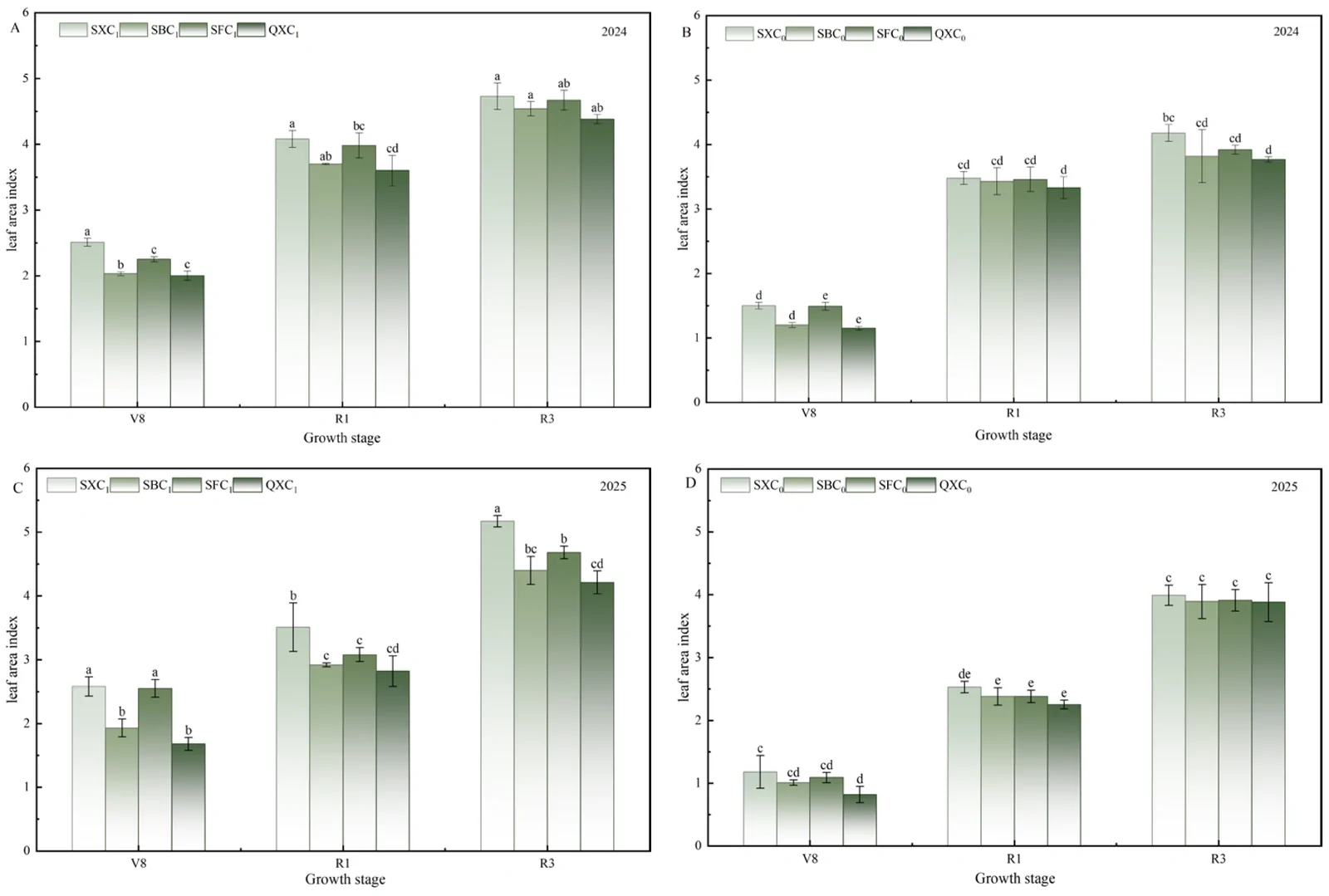
Leaf area index of maize under different tillage practices and plastic film mulching treatments. (**A**) Leaf area index under different tillage practices with plastic film mulching in 2024; (**B**) leaf area index under different tillage practices without plastic film mulching in 2024; (**C**) leaf area index under different tillage practices with plastic film mulching in 2025; (**D**) leaf area index under different tillage practices without plastic film mulching in 2025. Different lowercase letters indicate significant differences among tillage practices within the same growth stage at *p* < 0.05.

As shown in Figure 2, tillage practices and plastic film mulching significantly affected the leaf area index (LAI) of maize. In 2024, LAI gradually increased with crop development and reached a relatively high level at the R3 stage, whereas in 2025, LAI increased rapidly from V8 and peaked at R3. In both years, the mulched treatments (C_1_) generally exhibited higher LAI values than the corresponding non-mulched treatments (C_0_), with the promoting effect of mulching being more pronounced during the early growth stages. In 2024, LAI at the V8 stage was 51.01–73.91% higher under C_1_ than under C_0_, with significant differences among tillage practices (*p* < 0.05). At the R1 and R3 stages, the corresponding increases ranged from 7.87% to 17.24% and from 13.16% to 19.13%, respectively. In 2025, LAI at V8 increased by 91.09–133.94% under C_1_ and was significantly higher than that under C_0_ (*p* < 0.05). At R1, all mulched treatments also exhibited significantly higher LAI values than their corresponding non-mulched treatments (*p* < 0.05). At R3, SXC_1_ exhibited the highest LAI, with increases ranging from 22.69% to 38.74%. Among the tillage practices, SXC_1_ maintained relatively high LAI values at most growth stages.

### 2.3. Effects of Tillage Practices and Plastic Film Mulching on Maize Leaf Photosynthetic Characteristics

As shown in Table 1, Table 2, Table 3 and Table 4, tillage practices and plastic film mulching significantly affected maize leaf photosynthetic characteristics during 2024–2025, with deep rotary tillage combined with plastic film mulching (SXC_1_) generally showing superior performance. At the V8 stage, the net photosynthetic rate (*P_n_*) of SXC_1_ was 50.11 and 49.74 in 2024 and 2025, respectively, representing increases of 28.3% and 28.1% compared with deep rotary tillage without mulching (SXC_0_). At the R3 stage in 2024, *P_n_* under SXC_1_ was 34.5% higher than that under SXC_0_. The transpiration rate (*T_r_*) was generally higher under mulched than non-mulched treatments. Tillage practice had a highly significant effect on *T_r_* at all growth stages (*p* < 0.01), whereas the effect of plastic film mulching was significant only at the V8 stage (*p* < 0.05). Stomatal conductance (*G_s_*) also tended to be higher under mulched treatments, with SXC_1_ showing a 126.7% increase compared with SXC_0_ at the V8 stage in 2025. In contrast, intercellular CO_2_ concentration (*C_i_*) showed the opposite trend, decreasing by 26.2% and 13.0% at the V8 stage in 2024 and 2025, respectively (*p* < 0.05). Both plastic film mulching and tillage practice had highly significant effects on *P_n_*, *G_s_*, and *C_i_* at all growth stages (*p* < 0.01).

### 2.4. Effects of Tillage Practices and Plastic Film Mulching on Maize Dry Matter Accumulation

#### 2.4.1. Effects of Tillage Practices and Plastic Film Mulching on the Dynamics of Maize Dry Matter Accumulation

**Figure 3:**
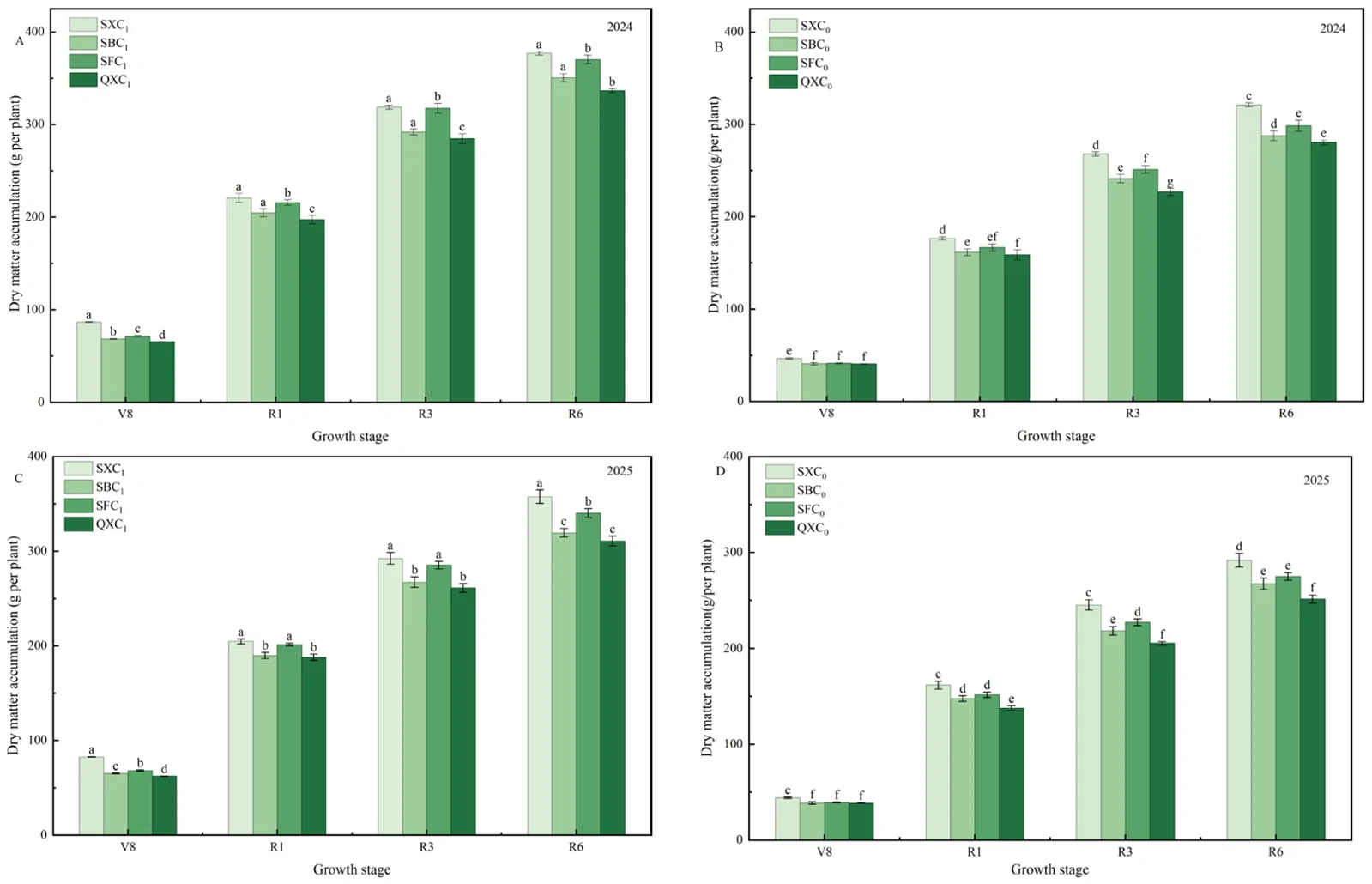
Dynamics of maize dry matter accumulation under different tillage practices and plastic film mulching treatments. (**A**) Dry matter accumulation dynamics under different tillage practices with plastic film mulching in 2024; (**B**) dry matter accumulation dynamics under different tillage practices without plastic film mulching in 2024; (**C**) dry matter accumulation dynamics under different tillage practices with plastic film mulching in 2025; (**D**) dry matter accumulation dynamics under different tillage practices without plastic film mulching in 2025. Different lowercase letters indicate significant differences among tillage practices within the same growth stage (p<0.05).

As shown in Figure 3, In both 2024 and 2025, maize dry matter accumulation increased progressively with crop development across all treatments and reached its maximum at the R6 stage. In 2024, dry matter accumulation under the mulched treatments (C_1_) was generally higher than that under the corresponding non-mulched treatments (C_0_) at all growth stages, with SXC_1_ exhibiting relatively high values at the V8, R1, and R3 stages. In 2025, the overall trend across treatments was consistent with that observed in 2024, with SXC_1_ maintaining relatively high dry matter accumulation at all growth stages. Compared with C_0_, C_1_ increased dry matter accumulation by 61.42–87.31% at V8, and by 24.44–36.52%, 19.00–27.21%, and 17.45–24.06% at R1, R3, and R6, respectively, indicating that the magnitude of the increase generally declined with crop development. Analysis of variance showed that year, tillage practice, and plastic film mulching had highly significant effects on dry matter accumulation at all growth stages (*p* < 0.001). The three-way interaction among year, tillage practice, and plastic film mulching was significant at V8 and R3 (*p* < 0.05), but was not significant at R1 or R6 (*p* > 0.05).

#### 2.4.2. Effects of Tillage Practices and Plastic Film Mulching on Post-Silking Dry Matter Accumulation in Maize

As shown in Table 5, Tillage practices and plastic film mulching significantly affected post-silking dry matter accumulation in maize. In 2024, compared with the corresponding non-mulched treatments, post-silking dry matter accumulation under deep rotary tillage with plastic film mulching (SXC_1_), deep harrowing with plastic film mulching (SBC_1_), deep plowing with plastic film mulching (SFC_1_), and subsoil rotary tillage with plastic film mulching (QXC_1_) increased by 8.07%, 15.87%, 17.03%, and 19.68%, respectively. Significant differences were observed between each mulched treatment and its corresponding non-mulched treatment (*p* < 0.05). Among the treatments, SXC_1_ showed a relatively high level of post-silking dry matter accumulation overall, whereas QXC_0_ was relatively low. In 2025, the overall trend was consistent with that observed in 2024. Compared with the corresponding C_0_ treatments, SXC_1_, SBC_1_, SFC_1_, and QXC_1_ increased post-silking dry matter accumulation by 17.44%, 8.22%, 12.66%, and 8.00%, respectively (*p* < 0.05), with SXC_1_ again maintaining a relatively high level. Analysis of variance showed that year, plastic film mulching, and tillage practice all had highly significant effects on post-silking dry matter accumulation (*p* < 0.01). The three-way interaction among year, plastic film mulching, and tillage practice was also highly significant (*p* < 0.01), indicating that the response of post-silking dry matter accumulation to different combinations of tillage practice and plastic film mulching varied significantly between years.

### 2.5. Effects of Tillage Practices and Plastic Film Mulching on Maize Yield and Yield-Related Traits

As shown in Table 6, plastic film mulching generally increased maize yield and yield-related traits in 2024. Compared with the corresponding C_0_ treatments, yield under C_1_ increased by 7.93–10.51%, while ear length, ear diameter, and 100-grain weight increased by 11.86–14.89%, 4.45–6.39%, and 8.25–11.78%, respectively. Among the treatments, SXC_1_ achieved the highest yield. The yields of SXC_1_, SBC_1_, and SFC_1_ were significantly higher than those of their corresponding C_0_ treatments (*p* < 0.05), whereas no significant difference was observed between QXC_1_ and QXC_0_ (*p* > 0.05). In 2025, the overall trend was consistent with that observed in 2024. Compared with C_0_, C_1_ increased yield by 4.94–10.27%, while ear length, ear diameter, and 100-grain weight increased by 11.18–20.73%, 2.77–11.79%, and 7.43–9.21%, respectively. SXC_1_ again achieved the highest yield, and the yields of SXC_1_ and SFC_1_ were significantly higher than those of their corresponding C_0_ treatments (*p* < 0.05). Analysis of variance showed that year, plastic film mulching, and tillage practice all had highly significant effects on yield (*p* < 0.01). The three-way interaction among year, plastic film mulching, and tillage practice had no significant effect on yield (*p* > 0.05), but significantly affected ear length (*p* < 0.05) and had a highly significant effect on 100-grain weight (*p* < 0.01).

### 2.6. Correlation Analysis

**Figure 4:**
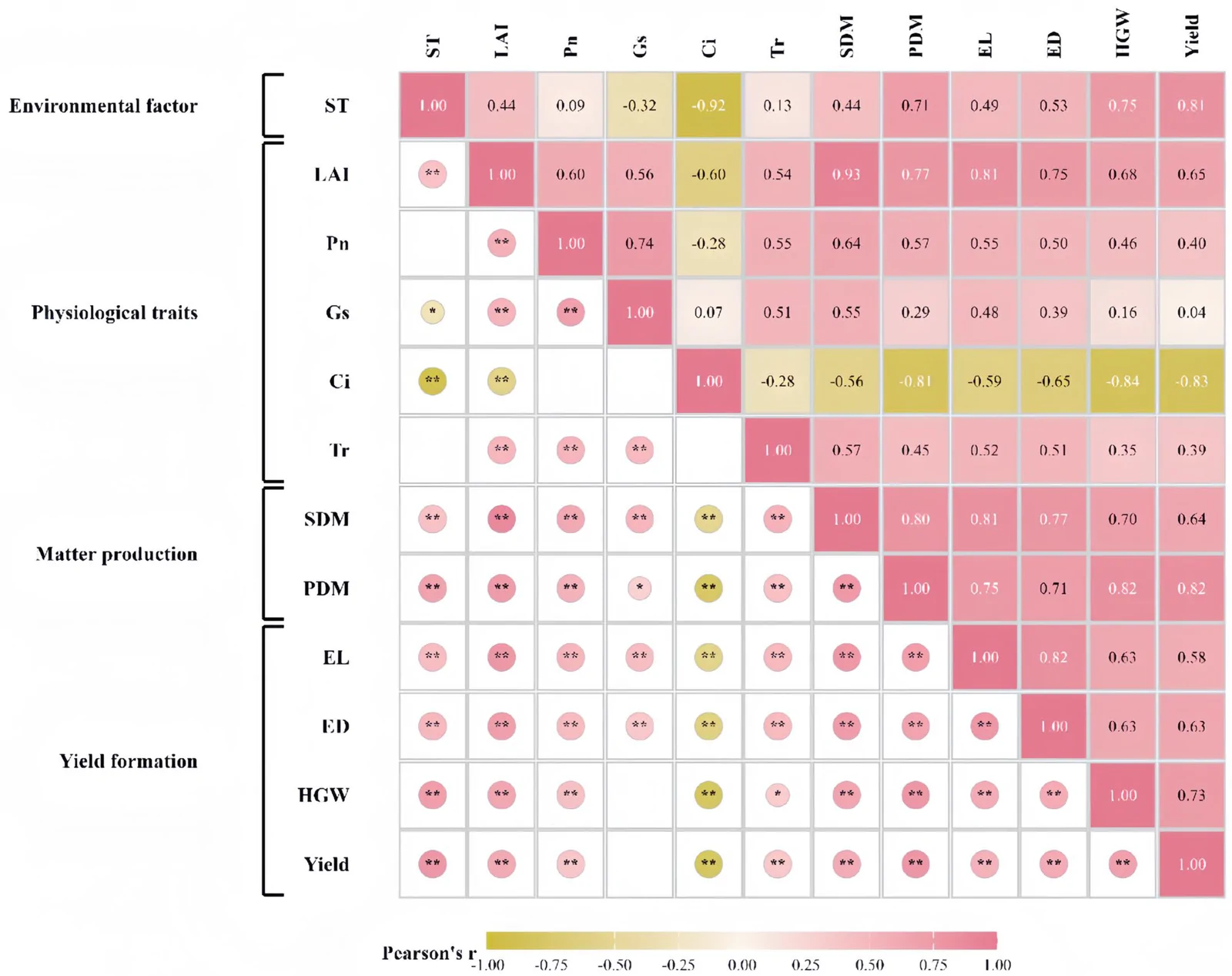
Correlation analysis among different indicators under different tillage practices and plastic film mulching treatments. * and ** indicate significant correlations at *p* < 0.05 and *p* < 0.01, respectively. ST, soil temperature; LAI, leaf area index; *P_n_*, net photosynthetic rate; *T_r_*, transpiration rate; *G_s_*, stomatal conductance; *C_i_*, intercellular CO_2_ concentration; SDM, dry matter accumulation; PDM, post-silking dry matter accumulation; EL, ear length; ED, ear diameter; HGW, 100-grain weight; Yield, yield.

As shown in Figure 4, yield formation was closely associated with dry matter accumulation. PDM was highly significantly and positively correlated with Yield (*p* < 0.01), while SDM and PDM generally showed significant or highly significant positive correlations with EL, ED, and HGW. Dry matter accumulation was also closely related to LAI and photosynthetic performance, with LAI showing a strong positive correlation with SDM. Strong positive correlations were also observed among *P_n_*, *T_r_*, and *G_s_*. Further analysis showed that ST was highly significantly and positively correlated with LAI, whereas *C_i_* was significantly negatively correlated with most indicators related to dry matter production and yield formation. Notably, ST was highly significantly and positively correlated with Yield (*p* < 0.01), indicating a close association between soil temperature and maize yield formation.

## 3. Discussion

In this study, plastic film mulching increased soil temperature during the maize growing season in both years, although the magnitude of warming varied markedly with growth stage, tillage practice, and year, indicating that the thermal effect of mulching was jointly regulated by soil conditions and meteorological factors. Plastic film mulching can improve the soil water and thermal environment in the cultivated layer by reducing evaporation and surface heat loss [11], which is particularly important in the cool regions of Northeast China, where spring warming is slow and effective accumulated temperature is limited [31]. Different tillage practices can influence soil heat transfer by altering soil bulk density, pore structure, and water distribution. Deeper soil disturbance can alleviate soil compaction and improve seedling growth [32], although its effectiveness is constrained by regional climatic and soil conditions [5]. In the present study, the tillage practice associated with the greatest warming effect was not consistent across growth stages or between 2024 and 2025, suggesting that no single tillage practice exhibited a consistently superior warming advantage. The warming effect of plastic film mulching was mainly concentrated during the early growth stages of maize and gradually weakened as the canopy developed and ambient temperature increased [33]. Increased precipitation during the growing season may also reduce the relative advantage of mulching [34], while under some conditions, fluctuations in soil moisture may exert a greater influence on maize production than changes in temperature [17]. It should be noted that soil temperature in this study was measured at fixed time intervals in the 0–5 cm surface soil layer. Therefore, the results mainly reflect the relative differences in surface soil temperature among treatments at specific measurement times and cannot fully characterize diurnal temperature variation, daily temperature extremes, or thermal conditions at different soil depths. In addition, soil moisture content was not measured simultaneously; therefore, changes in soil moisture among treatments could not be directly evaluated, nor could the relative contributions of temperature and moisture effects to maize growth and yield responses be quantitatively distinguished. Moreover, the integrity, damage rate, and degree of degradation of the PPC biodegradable film were not continuously monitored at different growth stages. Consequently, the influence of film degradation on the persistence of its soil-warming and moisture-conserving effects could not be quantitatively assessed. Future studies should integrate continuous temperature monitoring at multiple soil depths with simultaneous soil moisture measurements and strengthen the monitoring of film degradation dynamics to further elucidate the dynamic regulation of the root-zone water and thermal environment and associated crop responses under different tillage practices and plastic film mulching treatments.

The soil-warming effect induced by plastic film mulching may further promote canopy establishment and photosynthetic function. In this study, LAI was generally higher under mulched treatments than under non-mulched treatments, with the most pronounced response observed at the V8 stage. The values of *P_n_*, *T_r_*, and *G_s_* generally increased, whereas *C_i_* decreased, and SXC_1_ maintained relatively high levels at most growth stages. A larger leaf area is conducive to greater canopy light interception, and previous studies have shown that favorable water and thermal conditions in the rhizosphere can help maintain root activity and leaf photosynthetic capacity [21]. The coordinated regulation of soil moisture, temperature, and canopy radiation use by plastic film mulching may further promote dry matter production [35]. The increase in *P_n_* accompanied by a decrease in *C_i_* may reflect enhanced leaf CO_2_ assimilation capacity, while photosynthetic responses under mulching are closely associated with stomatal regulation [36]. Appropriate plastic film mulching may also help maintain relatively high photochemical efficiency [23]. Previous studies have further shown that tillage practices can influence aboveground physiological processes by modifying the space available for root growth as well as nutrient uptake and translocation [6], thereby further regulating dry matter accumulation in the grain [37]. Therefore, the overall advantage of SXC_1_ may not be attributable to the warming effect alone, but rather to the combined effects of deep rotary tillage and plastic film mulching on the soil environment, which may subsequently influence root growth, canopy establishment, and leaf photosynthetic processes. However, because soil bulk density, porosity, soil moisture, and root spatial distribution were not directly measured in this study, the relative contributions of these processes to the advantage of SXC_1_ cannot currently be quantitatively distinguished, and the underlying mechanisms require further verification.

The positive effects of plastic film mulching on canopy development and photosynthetic performance were further translated into advantages in dry matter production. In this study, dry matter accumulation was generally higher under mulched treatments at all growth stages, with the greatest increase observed at V8 and a gradually declining magnitude thereafter, indicating that the primary advantage of mulching was reflected in thermal compensation during the early growth stage and rapid vegetative establishment. In spring maize production in Northeast China, plastic film mulching can alter the pattern of dry matter accumulation by increasing effective thermal resources during the early growth period. Previous studies have reported that transparent plastic film can advance maize development by approximately 10 d, whereas black plastic film can advance development by 4–7 d depending on the year [38]. This is consistent with the greatest promotive effect of mulching on dry matter accumulation observed at V8 in the present study, suggesting that under cool conditions, the thermal compensation provided by plastic film mulching primarily favors rapid canopy establishment during vegetative growth. However, high yield formation depends not only on the biomass foundation established during the early growth period but also on sustained assimilation after silking. High-yielding maize requires a well-coordinated source–sink relationship [28], while the accumulation, partitioning, and remobilization of post-silking dry matter and nitrogen directly contribute to grain formation [39]. Moreover, as yield potential increases, the importance of newly assimilated dry matter after silking becomes increasingly pronounced [29]. In this study, post-silking dry matter accumulation increased under mulching across all four tillage practices, and SXC_1_ maintained relatively high levels in both years, indicating that its early growth advantage could be sustained into the reproductive stage. Final yield is jointly determined by dry matter production and redistribution within the crop population [40], and greater post-silking accumulation of dry matter and nitrogen is beneficial for maintaining kernel number and grain weight [30]. Therefore, the effects of plastic film mulching may not be limited to early-season soil warming, but may contribute to final yield formation through both rapid canopy establishment during the early growth stage and sustained dry matter production during the later growth period.

Plastic film mulching increased yield in both years, and SXC_1_ consistently achieved the highest yield, indicating that the combination of deep rotary tillage and plastic film mulching provided a relatively stable overall advantage. In 2024 and 2025, mulching increased yield by 7.93–10.51% and 4.94–10.27%, respectively, with SXC_1_ increasing yield by 10.51% and 10.27% compared with SXC_0_. In this study, the yield increase under mulching was consistent with improvements in soil warming, canopy development, and dry matter production. Previous studies have further shown that the yield-enhancing effect of plastic film mulching is also associated with soil moisture regulation, biomass production, and increased harvest index [24]. Long-term plastic film mulching can help sustain crop productivity by improving soil structure and water storage [41], while optimized water use in the root zone can also promote grain formation [42]. Yield responses vary among different mulching materials and mulching patterns [43], and other tillage and management practices within mulching systems in Northeast China may further regulate water use and yield responses [44]. Long-term tillage studies have shown that soil physical quality is an important basis for sustaining maize productivity [45]. Taken together, the yield advantage of SXC_1_ was more likely attributable to the combined effects of an improved soil thermal environment, enhanced canopy photosynthetic performance, and greater post-silking dry matter production, rather than to any single factor. Overall, SXC_1_ showed a favorable integrated performance under the conditions of this study. However, its stability across different climatic years and under long-term tillage conditions, as well as the specific contributions of soil physical conditions, root growth, and soil water processes, require further verification through multi-year long-term field experiments.

## 4. Materials and Methods

### 4.1. Experimental Site and Materials

**Figure 5:**
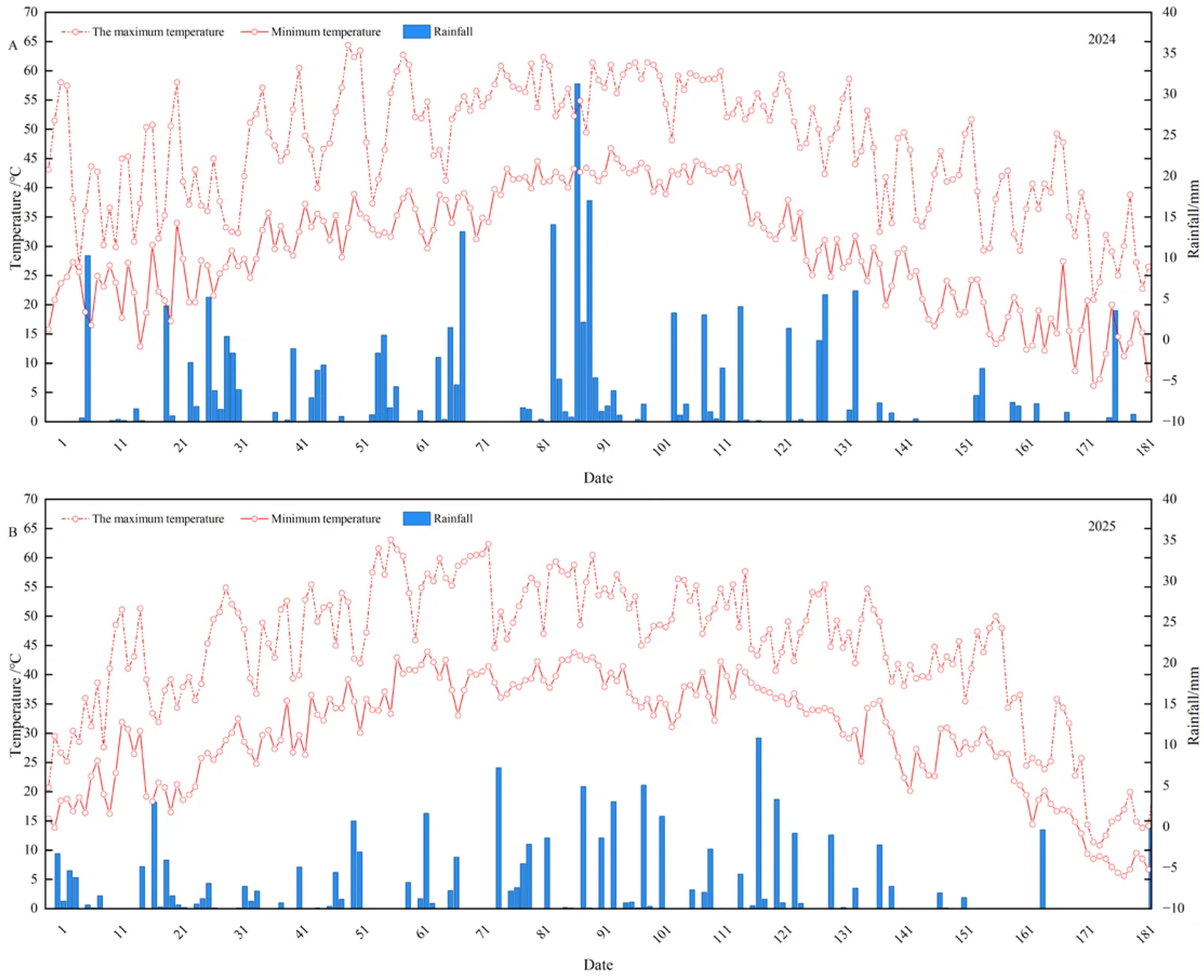
Dynamics of daily maximum temperature, daily minimum temperature, and daily precipitation at the experimental site from May to October in 2024 and 2025. (**A**) Dynamics of daily maximum temperature, daily minimum temperature, and daily precipitation at the experimental site from May to October 2024; (**B**) dynamics of daily maximum temperature, daily minimum temperature, and daily precipitation at the experimental site from May to October 2025.

A field experiment was conducted during 2024–2025 in Daxing Village, Hanzhang Township, Dunhua City, Yanbian Prefecture, Jilin Province, China (43.366920° N, 128.087764° E), with maize as the preceding crop. The region has a temperate continental climate, with a long-term mean annual precipitation of approximately 650 mm, an accumulated temperature of 2200–2300 °C, and a frost-free period of approximately 110 d. The dynamics of daily maximum temperature, daily minimum temperature, and daily precipitation during the crop growing seasons in 2024 and 2025 are shown in Figure 5. From May to October, the mean daily maximum temperatures were 22.72 and 21.10 °C in 2024 and 2025, respectively, while the mean daily minimum temperatures were 10.91 and 11.03 °C, respectively. Cumulative precipitation during the same period was 652.4 and 458.5 mm, respectively, indicating generally comparable thermal conditions between the two growing seasons but a marked interannual difference in precipitation. The planting density was 75,000 plants ha^−1^, with a row spacing of 60 cm and a plant spacing of 22 cm. The experimental soil was albic soil, and the main physicochemical properties of the 0–20 cm soil layer were as follows: organic matter, 42.71 g kg^−1^; pH, 5.35; available nitrogen, 242.03 mg kg^−1^; available phosphorus, 45.67 mg kg^−1^; available potassium, 157.7 mg kg^−1^; total nitrogen, 2.32 g kg^−1^; total phosphorus, 0.90 g kg^−1^; and total potassium, 19.71 g kg^−1^. The maize cultivar used was ‘Bulang 7’, provided by Beijing Zhongdi Seed Industry Co., Ltd. (Beijing, China). The maize cultivar used was ‘Bulang 7’. The biodegradable film was supplied by Jilin Difu Fertilizer Technology Co., Ltd., China (Changchun, Jilin Province, China), and was made of PPC, with a thickness of 0.008 mm and a width of 90 cm.

### 4.2. Experimental Design

A three-factor experimental design was used in this study. The plastic film mulching factor consisted of two levels: plastic film mulching (C_1_) and no mulching (C_0_). The tillage factor consisted of four practices: deep rotary tillage (SX), deep harrowing (SB), deep plowing (SF), and subsoil rotary tillage (QX). The combination of the two factors resulted in eight treatments: deep rotary tillage with plastic film mulching (SXC_1_), deep harrowing with plastic film mulching (SBC_1_), deep plowing with plastic film mulching (SFC_1_), subsoil rotary tillage with plastic film mulching (QXC_1_), deep rotary tillage without mulching (SXC_0_), deep harrowing without mulching (SBC_0_), deep plowing without mulching (SFC_0_), and subsoil rotary tillage without mulching (QXC_0_). Each treatment was replicated three times. For the SX treatment, deep rotary tillage was performed to a depth of 20 cm. For the SB treatment, deep harrowing was conducted to a depth of 15 cm. For the SF treatment, deep plowing was performed to a depth of 25 cm. For the QX treatment, subsoil rotary tillage was conducted to loosen the deeper soil layer without soil inversion, thereby improving soil structure while maintaining the original soil-layer arrangement. For the mulched treatments, the PPC biodegradable film described above was mechanically laid to continuously cover the entire ridge surface, with both edges secured with soil. No film was applied in the non-mulched treatments. Maize was sown on 4 May 2024 and 8 May 2025 and harvested on 27 October 2024 and 29 October 2025, respectively. The experimental plots were randomly arranged, with guard rows established around the plots. Except for tillage practice and plastic film mulching, all treatments received the same planting density, fertilization regime, and field management practices, including pest, disease, and weed control, to minimize the influence of factors other than the experimental treatments.

### 4.3. Measurements and Methods

#### 4.3.1. Soil Temperature

To compare the relative differences in surface soil temperature among treatments, soil temperature was monitored at a fixed time during the maize growing season. Measurements began on 14 May 2024 and 18 May 2025, respectively. The 0–5 cm soil layer between maize rows was selected as the measurement position, and soil temperature was subsequently measured every 10 d at 11:00 a.m. During each measurement, three representative points were randomly selected, and a soil thermometer was inserted into the 0–5 cm soil layer. The temperature was recorded after the reading had stabilized, and the mean value of the three measurement points was used for statistical analysis. To evaluate the soil-warming effect of plastic film mulching, the warming magnitude was expressed as the difference in soil temperature between C_1_ and C_0_ under the same tillage practice and was calculated using the following equation:

Soil temperature increase:

(1) $$\Delta T=T_{C_{1}}-T_{C_{0}}$$

where ΔT represents the soil temperature increase (°C), $T_{C_{1}}$ represents the soil temperature under film mulching (C_1_) across different tillage modes, and $T_{C_{0}}$ represents the soil temperature without film mulching (C_0_).

#### 4.3.2. Leaf Area Index

Leaf area index (LAI) was determined using the leaf length–width method at the V8, R1, and R3 growth stages. For each treatment, three intact maize plants were randomly selected, and the length (L) and maximum width (W) of all fully expanded leaves on each plant were measured using a measuring tape. The area of an individual leaf was calculated as: LA (cm^2^ plant^−1^) = L × W × 0.75, and; the leaf area index was then calculated as:

LAI=(∑LA×N)/A,

where LA is the leaf area per plant, N is the number of plants within the ground area S, and S is the corresponding ground area.

Individual leaf area (LA):

(2) LA=L×W×0.75

Leaf area index (LAI):

(3) $$LAI=\frac{\sum LA\times N}{A}$$

where LA is the individual leaf area (cm^2^), L is the leaf length (cm), W is the maximum leaf width (cm), LAI is the leaf area index, TLA is the total leaf area per plant (cm^2^ plant^−1^), N is the number of plants within the sampled ground area, and S is the sampled ground area (cm^2^). The coefficient 0.75 is an empirical correction factor for maize leaf area.

#### 4.3.3. Photosynthetic Characteristics

Leaf gas-exchange parameters were measured in the field at approximately 11:00 a.m. under clear weather conditions. For each treatment, three representative plants with uniform growth and no visible symptoms of pests or diseases were randomly selected. At the V8 stage, the uppermost fully expanded leaf was selected for measurement, whereas the ear leaf was measured at the R1 and R3 stages. Leaf net photosynthetic rate (*P_n_*), stomatal conductance (*G_s_*), transpiration rate (*T_r_*), and intercellular CO_2_ concentration (*C_i_*) were measured using a LI-6800 portable photosynthesis system (LI-COR Biosciences, Lincoln, NE, USA). During measurements, the photosynthetic photon flux density (PPFD) in the leaf chamber was set to 1500 μmol m^−2^ s^−1^, the reference CO_2_ concentration was maintained at 400 μmol mol^−1^, and the flow rate was set to 500 μmol s^−1^. Leaf chamber temperature and relative humidity were not artificially controlled and therefore varied with ambient field conditions. Treatments were measured consecutively in the order SXC_1_, SBC_1_, SFC_1_, QXC_1_, SXC_0_, SBC_0_, SFC_0_, and QXC_0_, and all measurements were completed within as short a period as possible to minimize the influence of environmental variation during the measurement period. Data were recorded after the leaf gas-exchange parameters had stabilized.

#### 4.3.4. Dry Matter Accumulation

Dry matter accumulation was determined at the V8, R1, R3, and physiological maturity (R6) stages. For each treatment, three uniformly growing and representative plants were randomly sampled from each plot and placed separately in mesh bags. The fresh weight of each plant was recorded immediately after sampling. The samples were then oven-dried at 105 °C for 1 h to deactivate enzymatic activity and subsequently dried at 80 °C to a constant weight. The dry weight of each plant was then recorded. Post-silking dry matter accumulation was calculated according to the following equation:

Post-silking dry matter accumulation:

(4) $$PDM=X_{6}-X_{1}$$

where PDM represents post-silking dry matter accumulation (g plant^−1^), X_6_ represents dry matter accumulation at the R6 stage (physiological maturity) (g plant^−1^), and X_1_ represents dry matter accumulation at the R1 stage (silking) (g plant^−1^).

#### 4.3.5. Yield and Yield-Related Traits

At the R6 stage, three representative sampling areas with uniform plant growth and no missing plants were selected within each plot, avoiding the border rows and both ends of the plot. Each sampling area was 10 m^2^. All ears within each sampling area were harvested and weighed, and the number of effective ears was recorded. Ten ears were then randomly selected and air-dried naturally before threshing. Ear length, ear diameter, and 100-grain weight were measured, and grain moisture content was determined simultaneously. Yield for each plot was adjusted to a standard grain moisture content of 14% and converted to yield per unit area according to the actual harvested area.

### 4.4. Data Processing and Statistical Analysis

All experimental data were organized and preliminarily processed using Microsoft Excel 2019 (Microsoft Corporation, Redmond, WA, USA), and data presented in the figures and tables represent the mean of three replicates. Statistical analyses were performed using IBM SPSS Statistics 20.0 (IBM Corp., Armonk, NY, USA). One-way analysis of variance (ANOVA), followed by Duncan’s multiple range test, was used to determine significant differences among treatments, with the significance level set at *p* < 0.05. Two-way ANOVA was used to evaluate the effects of tillage practice, plastic film mulching, and their interaction on soil temperature, leaf area index (LAI), photosynthetic parameters, aboveground dry matter accumulation (SDM), and yield components. All statistical analyses were based on three independent replicates. Data visualization and graphical presentation were performed using OriginPro 2026 (OriginLab Corporation, Northampton, MA, USA) and R (version 4.5.3), with correlation analysis and heatmap visualization conducted in R.

## 5. Conclusions

Two consecutive years of field experiments showed that plastic film mulching increased surface soil temperature during the maize growing season in a cool region and promoted leaf area index (LAI), leaf photosynthetic performance, dry matter accumulation, and post-silking dry matter accumulation, ultimately increasing yield. The responses to plastic film mulching differed markedly among tillage practices, with deep rotary tillage combined with plastic film mulching (SXC_1_) showing a relatively stable overall advantage. Compared with deep rotary tillage without mulching (SXC_0_), SXC_1_ increased the net photosynthetic rate at the V8 stage by 28.3% and 28.1% in 2024 and 2025, respectively, post-silking dry matter accumulation by 8.07% and 17.44%, respectively, and yield by 10.51% and 10.27%, respectively. Moreover, SXC_1_ consistently achieved the highest yield in both years. Overall, under the cool ecological conditions of this study, the combination of deep rotary tillage and plastic film mulching was conducive to coordinating early canopy establishment, photosynthetic performance, and post-silking dry matter production and may serve as a potential management practice for high-yield and resource-efficient maize production in this region. However, its long-term applicability requires further evaluation through multi-year field experiments.

## Figures and Tables

**Table 1:** Net photosynthetic rate and analysis of variance of maize leaves under different tillage practices and plastic film mulching.

Year	Target	Treatment	V8	R1	R3
2024	*P_n_* [μmol/(m^2^·s)]	SXC_1_	50.11 ± 1.58 a	35.29 ± 5.89 a	43.93 ± 3.33 a
		SBC_1_	46.54 ± 1.49 a	33.27 ± 5.15 a	37.15 ± 3.92 ab
		SFC_1_	48.73 ± 5.79 ab	34.36 ± 4.80 a	41.13 ± 1.49 bc
		QXC_1_	44.02 ± 1.46 ab	32.68 ± 2.72 ab	35.07 ± 4.06 bcd
		SXC_0_	39.06 ± 5.76 bc	31.61 ± 4.30 ab	32.67 ± 5.26 cd
		SBC_0_	32.23 ± 5.49 bc	28.41 ± 4.31 ab	31.72 ± 1.15 cd
		SFC_0_	38.32 ± 6.33 c	29.34 ± 5.79 ab	31.96 ± 0.79 cd
		QXC_0_	31.06 ± 4.54 c	24.22 ± 2.34 b	30.07 ± 2.21 d
2025	*P_n_* [μmol/(m^2^·s)]	SXC_1_	49.74 ± 2.40 a	42.47 ± 3.84 a	37.65 ± 0.68 a
		SBC_1_	45.39 ± 4.42 ab	33.46 ± 5.24 bc	35.31 ± 3.07 a
		SFC_1_	47.04 ± 1.20 a	37.78 ± 4.05 ab	36.65 ± 4.27 a
		QXC_1_	45.29 ± 4.89 ab	33.17 ± 5.22 bc	32.92 ± 4.90 ab
		SXC_0_	38.83 ± 5.80 b	31.64 ± 3.29 bcd	32.46 ± 0.42 ab
		SBC_0_	30.72 ± 1.65 c	28.77 ± 5.52 cd	29.66 ± 1.69 b
		SFC_0_	31.40 ± 4.91 c	31.51 ± 4.3 bcd	32.19 ± 1.67 ab
		QXC_0_	30.61 ± 3.00 c	24.52 ± 1.77 d	29.64 ± 1.76 b
Year (Y)			NS	NS	*
Film coverage factor (C)			**	**	**
Tillage practice (F)			**	**	**
Y × C × F			NS	NS	NS

Note: Data are presented as the mean ± standard deviation (SD). Within the same year and growth stage, different lowercase letters indicate significant differences among treatments (*p* < 0.05). * and ** indicate significance at *p* < 0.05 and *p* < 0.01, respectively; NS indicates no significant difference. Y, C, and F represent year, film coverage factor, and tillage practice, respectively; Y × C × F represents the interaction among year, film coverage factor, and tillage practice.

**Table 2:** Transpiration rate and analysis of variance of maize leaves under different tillage practices and plastic film mulching.

Year	Target	Treatment	V8	R1	R3
2024	*T_r_* [mmol/(m^2^·s)]	SXC_1_	9.80 ± 1.75 a	7.12 ± 1.77 a	9.40 ± 1.02 a
		SBC_1_	8.32 ± 1.89 a	6.85 ± 0.49 a	9.03 ± 1.24 a
		SFC_1_	9.60 ± 1.69 a	7.03 ± 1.24 a	9.26 ± 0.13 a
		QXC_1_	7.89 ± 0.71 ab	6.31 ± 1.06 a	8.31 ± 1.13 ab
		SXC_0_	5.52 ± 0.82 bc	6.01 ± 0.96 a	6.82 ± 0.75 bc
		SBC_0_	4.03 ± 0.44 c	5.81 ± 0.46 a	6.24 ± 1.08 c
		SFC_0_	5.04 ± 0.66 c	5.90 ± 0.43 a	6.32 ± 0.37 c
		QXC_0_	3.33 ± 1.01 c	5.54 ± 0.79 a	6.08 ± 0.35 c
2025	*T_r_* [mmol/(m^2^·s)]	SXC_1_	9.6 ± 1.69 a	7.7 ± 0.89 a	9.32 ± 1.12 a
		SBC_1_	8.28 ± 1.71 ab	6.68 ± 1.04 a	8.48 ± 1.24 ab
		SFC_1_	8.8 ± 3.23 a	7.22 ± 1.79 a	8.68 ± 0.43 ab
		QXC_1_	6.89 ± 0.5 abc	6.4 ± 1.03 a	8.38 ± 0.28 ab
		SXC_0_	5.52 ± 0.82 bc	6.31 ± 0.66 a	7.42 ± 0.80 bcd
		SBC_0_	4.47 ± 0.1 c	5.77 ± 0.55 a	6.02 ± 0.11 d
		SFC_0_	4.62 ± 1.27 c	5.84 ± 1.09 a	6.62 ± 1.21 cd
		QXC_0_	4.38 ± 0.16 c	5.49 ± 0.97 a	5.90 ± 1.65 d
Year (Y)			NS	NS	NS
Film coverage factor (C)			*	NS	NS
Tillage practice (F)			**	**	**
Y × C × F			NS	NS	NS

Note: Data are presented as the mean ± standard deviation (SD). Within the same year and growth stage, different lowercase letters indicate significant differences among treatments (*p* < 0.05). * and ** indicate significance at *p* < 0.05 and *p* < 0.01, respectively; NS indicates no significant difference. Y, C, and F represent year, film coverage factor, and tillage practice, respectively; Y × C × F represents the interaction among year, film coverage factor, and tillage practice.

**Table 3:** Stomatal conductance and analysis of variance of maize leaves under different tillage practices and plastic film mulching.

Year	Target	Treatment	V8	R1	R3
2024	*G_s_* [mmol/(m^2^·s)]	SXC_1_	0.20 ± 0.04 a	0.14 ± 0.04 a	0.24 ± 0.07 a
		SBC_1_	0.16 ± 0.08 ab	0.13 ± 0.03 a	0.18 ± 0.01 ab
		SFC_1_	0.19 ± 0.09 abc	0.14 ± 0.04 a	0.22 ± 0.04 abc
		QXC_1_	0.14 ± 0.01 abc	0.12 ± 0.02 a	0.16 ± 0.04 bc
		SXC_0_	0.10 ± 0.02 abc	0.12 ± 0.02 a	0.16 ± 0.01 bc
		SBC_0_	0.08 ± 0.01 bc	0.11 ± 0.01 a	0.15 ± 0.01 c
		SFC_0_	0.10 ± 0.03 c	0.11 ± 0.03 a	0.15 ± 0.03 c
		QXC_0_	0.08 ± 0.05 c	0.10 ± 0.01 a	0.14 ± 0.04 c
2025	*G_s_* [mmol/(m^2^·s)]	SXC_1_	0.34 ± 0.09 a	0.29 ± 0.06 a	0.26 ± 0.02 a
		SBC_1_	0.19 ± 0.09 b	0.23 ± 0.05 ab	0.20 ± 0.03 b
		SFC_1_	0.2 ± 0.04 b	0.26 ± 0.03 a	0.25 ± 0.01 a
		QXC_1_	0.16 ± 0.06 bc	0.17 ± 0.01 bc	0.18 ± 0.01 bc
		SXC_0_	0.15 ± 0.05 bc	0.15 ± 0.02 c	0.16 ± 0.04 bc
		SBC_0_	0.1 ± 0.02 bc	0.12 ± 0.02 c	0.15 ± 0.03 bc
		SFC_0_	0.14 ± 0.01 bc	0.13 ± 0.01 c	0.16 ± 0.01 bc
		QXC_0_	0.08 ± 0.09 c	0.11 ± 0.01 c	0.14 ± 0.04 c
Year (Y)			*	**	NS
Film coverage factor (C)			**	**	**
Tillage practice (F)			**	**	**
Y × C × F			NS	NS	NS

Note: Data are presented as the mean ± standard deviation (SD). Within the same year and growth stage, different lowercase letters indicate significant differences among treatments (*p* < 0.05). * and ** indicate significance at *p* < 0.05 and *p* < 0.01, respectively; NS indicates no significant difference. Y, C, and F represent year, film coverage factor, and tillage practice, respectively; Y × C × F represents the interaction among year, film coverage factor, and tillage practice.

**Table 4:** Intercellular CO_2_ concentration and analysis of variance of maize leaves under different tillage practices and plastic film mulching.

Year	Target	Treatment	V8	R1	R3
2024	*C_i_* (μmol/mol)	SXC_1_	129.01 ± 9.09 e	94.11 ± 7.01 d	134.88 ± 4.71 e
		SBC_1_	160.81 ± 5.18 e	100.89 ± 7.95 cd	165.62 ± 9.36 d
		SFC_1_	134.17 ± 8.92 d	95.68 ± 4.80 bcd	161.27 ± 3.52 cd
		QXC_1_	172.32 ± 7.10 cd	104.56 ± 4.59 bcd	171.90 ± 7.52 bcd
		SXC_0_	174.91 ± 4.90 c	106.42 ± 3.79 bc	173.38 ± 8.38 bcd
		SBC_0_	264.30 ± 5.58 b	132.56 ± 5.88 b	179.43 ± 6.45 bc
		SFC_0_	233.59 ± 9.02 a	109.12 ± 7.01 a	174.44 ± 5.53 ab
		QXC_0_	269.38 ± 10.10 a	137.97 ± 3.62 a	189.49 ± 4.27 a
2025	*C_i_* (μmol/mol)	SXC_1_	186.37 ± 8.30 g	131.56 ± 2.07 f	150.53 ± 5.62 e
		SBC_1_	199.71 ± 7.11 ef	147.31 ± 4.79 de	159.94 ± 7.22 de
		SFC_1_	193.31 ± 4.46 fg	139.75 ± 5.35 ef	154.71 ± 4.23 e
		QXC_1_	206.22 ± 6.09 de	151.88 ± 3.24 cd	166.71 ± 6.81 cd
		SXC_0_	214.32 ± 5.15 cd	159.78 ± 7.70 c	173.95 ± 5.56 c
		SBC_0_	229.58 ± 5.81 ab	177.23 ± 6.18 ab	188.90 ± 4.55 ab
		SFC_0_	223.14 ± 6.22 bc	169.27 ± 5.70 b	183.46 ± 2.40 b
		QXC_0_	239.21 ± 5.88 a	184.79 ± 5.53 a	195.44 ± 4.50 a
Year (Y)			**	**	NS
Film coverage factor (C)			**	**	**
Tillage practice (F)			**	**	**
Y × C × F			**	NS	**

Note: Data are presented as the mean ± standard deviation (SD). Within the same year and growth stage, different lowercase letters indicate significant differences among treatments (*p* < 0.05). ** The difference reached a statistically significant level of *p* < 0.01; NS indicates no significant difference. Y, C, and F represent year, film coverage factor, and tillage practice, respectively; Y × C × F represents the interaction among year, film coverage factor, and tillage practice.

**Table 5:** Post-silking dry matter accumulation and analysis of variance in maize under different tillage practices and plastic film mulching.

Year	Treatment	X_1_	X_6_	PDM
2024	SXC_1_	220.73 ± 4.84 a	377.00 ± 2.03 a	156.27 ± 2.82 a
	SBC_1_	204.47 ± 4.27 a	350.47 ± 4.54 a	146.00 ± 0.82 a
	SFC_1_	215.80 ± 2.92 b	370.20 ± 4.63 b	154.40 ± 4.07 b
	QXC_1_	197.27 ± 4.58 c	336.60 ± 2.18 b	145.73 ± 3.09 b
	SXC_0_	176.40 ± 1.67 d	321.00 ± 2.21 c	144.60 ± 3.44 b
	SBC_0_	161.50 ± 3.60 e	287.50 ± 5.19 d	126.00 ± 3.64 c
	SFC_0_	166.47 ± 3.93 ef	298.40 ± 6.10 e	131.93 ± 4.81 d
	QXC_0_	158.53 ± 5.39 f	280.30 ± 2.55 e	121.77 ± 3.20 d
2025	SXC_1_	204.55 ± 2.64 a	357.58 ± 7.21 a	153.02 ± 4.57 a
	SBC_1_	189.71 ± 3.29 b	319.45 ± 4.67 c	129.73 ± 1.58 c
	SFC_1_	201.21 ± 1.45 a	340.20 ± 4.85 b	138.99 ± 3.43 b
	QXC_1_	187.78 ± 3.30 b	310.74 ± 5.25 c	122.96 ± 2.12 c
	SXC_0_	161.58 ± 4.10 c	291.88 ± 7.07 d	130.30 ± 2.97 d
	SBC_0_	147.47 ± 2.94 d	267.35 ± 5.94 e	119.88 ± 3.03 d
	SFC_0_	151.51 ± 2.79 d	274.88 ± 4.07 e	123.37 ± 1.36 d
	QXC_0_	137.55 ± 2.42 e	251.40 ± 4.22 f	113.85 ± 2.30 e
Year (Y)		**	**	**
Film coverage factor (C)		**	**	**
Tillage practice (F)		**	**	**
Y × C × F		NS	NS	**

Note: X_1_ and X_6_ represent dry matter accumulation at the R1 and R6 stages, respectively; PDM represents post-silking dry matter accumulation and was calculated as X_6_ − X_1_. Data are presented as mean ± standard deviation. Different lowercase letters within the same year and growth stage indicate significant differences among treatments (*p* < 0.05). ** indicates significance at *p* < 0.01, and NS indicates no significant difference. Y, C, and F represent year, film coverage, and tillage mode, respectively, while Y × C × F represents the interaction among year, film coverage, and tillage mode.

**Table 6:** Maize yield, yield-related traits, and analysis of variance under different tillage practices and plastic film mulching.

Year	Treatment	Yield (kg·hm^−2^)	Ear Length (cm)	Ear Diameter (cm)	100-Grain Weight (g)
2024	SXC_1_	12,254.00 ± 480.27 a	23.23 ± 0.74 a	5.16 ± 0.08 a	34.34 ± 0.74 a
	SBC_1_	12,108.22 ± 697.16 a	21.83 ± 0.67 ab	4.93 ± 0.12 ab	32.43 ± 0.16 b
	SFC_1_	12,160.59 ± 377.83 ab	22.63 ± 0.15 bc	5.04 ± 0.11 abc	33.23 ± 0.56 b
	QXC_1_	11,271.92 ± 199.54 bc	21.10 ± 0.44 cd	4.90 ± 0.05 bc	31.36 ± 0.68 c
	SXC_0_	11,089.06 ± 572.05 c	20.60 ± 0.10 d	4.85 ± 0.13 bcd	30.72 ± 0.18 cd
	SBC_0_	10,967.79 ± 182.46 c	19.00 ± 0.44 d	4.72 ± 0.03 cd	29.92 ± 0.72 cd
	SFC_0_	11,038.17 ± 254.26 c	20.23 ± 0.35 e	4.75 ± 0.05 cd	30.38 ± 0.50 d
	QXC_0_	10,443.26 ± 448.40 c	18.83 ± 0.67 e	4.67 ± 0.23 d	28.97 ± 0.47 e
2025	SXC_1_	11,099.83 ± 301.37 a	22.37 ± 0.58 a	5.01 ± 0.15 a	31.51 ± 0.54 a
	SBC_1_	10,596.20 ± 28.93 abc	21.78 ± 0.86 ab	4.93 ± 0.29 ab	29.72 ± 0.44 b
	SFC_1_	11,004.99 ± 311.63 ab	22.00 ± 0.14 a	5.00 ± 0.08 a	30.79 ± 0.27 a
	QXC_1_	10,085.43 ± 282.44 bcd	20.73 ± 0.38 abc	4.83 ± 0.17 abc	29.40 ± 0.19 b
	SXC_0_	10,065.70 ± 326.35 bcd	20.12 ± 1.01 bc	4.70 ± 0.03 bc	29.33 ± 0.48 b
	SBC_0_	9739.43 ± 338.82 cd	18.04 ± 1.25 d	4.41 ± 0.12 d	27.46 ± 0.62 d
	SFC_0_	10,012.29 ± 834.51 cd	19.78 ± 0.37 c	4.59 ± 0.11 cd	28.28 ± 0.50 c
	QXC_0_	9610.82 ± 815.03 d	18.02 ± 1.40 d	4.70 ± 0.03 bc	26.92 ± 0.52 d
Year (Y)		**	**	**	**
Film coverage factor (C)		**	**	*	**
Tillage practice (F)		**	**	**	**
Y × C × F		NS	*	NS	**

Note: The data in this table represent the mean ± standard deviation. Different lowercase letters among treatments within the same year and same planting period indicate significant differences (*p* < 0.05); *denotes a significance level of *p* < 0.05; ** denotes a significance level of *p* < 0.01; NS indicates no significant difference; Y, C, and F represent year, mulching factor, and cropping system, respectively; Y × C × F denotes the interaction among year, mulching factor, and cropping system.

## Data Availability

All relevant data are contained within the manuscript.
